# Hematological Inflammatory Indices and Coagulation Parameters in Individuals with Substance Use Disorder: A Comparative Study with Healthy Controls

**DOI:** 10.3390/biomedicines14051000

**Published:** 2026-04-28

**Authors:** Fatih Saglam, Bilal Altunisik, Ahmet Yaltir, Necip Nas

**Affiliations:** 1 Department of Psychiatry, Medical Faculty, Adıyaman University, 02040 Adıyaman, Türkiye; 2 Department of Internal Medicine, Medical Faculty, Siirt University, 56100 Siirt, Türkiye; baltunisik@gmail.com (B.A.); necipnas@gmail.com (N.N.); 3 Department of Pharmacy Biochemistry, Pharmacy Faculty, Mersin University, 33110 Mersin, Türkiye; yaltir_ahmet@mersin.edu.tr

**Keywords:** substance use disorder, inflammation, hematological indices, platelet activation, coagulation parameters, D-dimer, biomarkers

## Abstract

**Background:** Substance use disorder (SUD) is associated with systemic inflammatory activation and may influence hematological and coagulation-related pathways. Chronic exposure to psychoactive substances may promote immune activation, endothelial dysfunction, and increased platelet reactivity, thereby contributing to inflammation–coagulation crosstalk. Routine laboratory markers derived from complete blood count and coagulation tests have recently gained attention as potential indicators of these biological alterations. **Objective:** This study aimed to evaluate hematological inflammatory indices, platelet-related parameters, and coagulation markers in individuals with SUD compared with healthy controls. **Methods:** This cross-sectional study included individuals diagnosed with SUD (*n* = 55) and age- and sex-matched healthy controls (*n* = 40). Hematological parameters, including neutrophil, lymphocyte, monocyte, platelet indices, and derived inflammatory ratios, were analyzed. Coagulation parameters, including prothrombin time (PT), activated partial thromboplastin time (aPTT), international normalized ratio (INR), fibrinogen, and D-dimer levels, were also evaluated. Statistical analyses were performed to compare laboratory findings between groups. **Results:** Significant alterations were observed in several hematological and fibrinolytic parameters in individuals with SUD. Specifically, the neutrophil-to-lymphocyte ratio (NLR), platelet count (PLT), and D-dimer levels were significantly higher in the study group compared with the controls, suggesting increased inflammatory activity, platelet activation, and fibrinolysis. In contrast, the fibrinogen levels did not show clinically meaningful differences between the groups. **Conclusions:** Individuals with SUD exhibit alterations in hematological inflammatory indices, platelet-related parameters, and fibrinolytic markers. Routine hematological and coagulation parameters may provide accessible indicators of systemic inflammatory and hemostatic disturbances associated with SUD and may support future research aimed at identifying potential biological markers of addiction.

## 1. Introduction

Substance use has accompanied human societies for centuries and has been practiced for various purposes, including recreational, religious, and perceived therapeutic uses [1]. However, repeated exposure to psychoactive substances may lead to psychological and physical dependence, resulting in substance use disorder (SUD) [2]. SUD is recognized as a chronic, relapsing condition characterized by compulsive use, loss of control, and continued use despite harm [3].

In contemporary diagnostic frameworks, SUD has replaced earlier classifications such as abuse and dependence, reflecting the dimensional nature of substance-related pathology [4]. A wide range of substances including alcohol, cannabinoids, opioids, benzodiazepines, and stimulants are associated with distinct neurobiological and clinical profiles [5]. SUDs represent a major global health burden, contributing significantly to morbidity and mortality [6,7].

Growing evidence indicates that inflammatory processes play a key role in the pathophysiology of addiction [8]. Chronic substance exposure activates neuroimmune pathways, leading to neuroinflammation and systemic inflammatory responses that may contribute to structural and functional brain alterations [9,10]. Inflammation is closely linked to hemostasis, as inflammatory mediators promote endothelial dysfunction, platelet activation, and coagulation cascade activation, resulting in a prothrombotic state (thromboinflammation) [11,12,13]. Endothelial injury, platelet reactivity, and altered fibrinolysis represent key components of this process. Substance use, particularly of stimulants and opioids, has been associated with oxidative stress, vascular injury, and coagulation dysregulation, further supporting the link between SUD and hemostatic imbalance [14,15].

Peripheral inflammatory biomarkers have gained attention as accessible indicators of these processes. Hematological indices derived from complete blood count (CBC), such as neutrophil-to-lymphocyte ratio (NLR), platelet-to-lymphocyte ratio (PLR), and monocyte-to-lymphocyte ratio (MLR), are widely used markers of systemic inflammation [16,17,18]. Importantly, different substances exert distinct immunological and hematological effects. Stimulants are associated with sympathetic activation and endothelial injury, whereas opioids modulate immune responses through receptor-mediated mechanisms. Cannabis may differentially affect the platelet function, cytokine release, and coagulation pathways, highlighting the heterogeneity of biological responses across substances [19,20,21].

Recent studies have explored CBC-derived indices as potential biomarkers in SUD [19,22]. In addition to CBC-derived markers, coagulation parameters such as prothrombin time (PT), activated partial thromboplastin time (aPTT), fibrinogen, and D-dimer provide complementary information on hemostatic and fibrinolytic activity. D-dimer reflects fibrin degradation, while fibrinogen acts as both an acute-phase reactant and a key coagulation factor, supporting their role in assessing inflammation–coagulation interactions [12,23].

Despite these findings, the role of peripheral hematological and coagulation markers in SUD remains incompletely understood, with most studies focusing on single markers or specific substances. Therefore, the present study aims to provide an integrated evaluation of hematological inflammatory indices, platelet-related parameters, and coagulation/fibrinolytic markers in individuals with SUD. We hypothesized that patients with SUD would exhibit elevated systemic inflammatory and hemostatic alterations compared with. the healthy controls.

## 2. Materials and Method

### 2.1. Ethics Statement

This retrospective study was conducted in accordance with the ethical principles of the Declaration of Helsinki. Ethical approval was obtained from the Siirt University Ethics Committee (Decision No.: 2023/12/01/02, Date: 29 December 2023). Due to the retrospective design of the study, the requirement for written informed consent was waived by the ethics committee. All patient data were anonymized prior to analysis, and confidentiality was strictly maintained throughout the study.

### 2.2. Study Design and Participants

This comparative cross-sectional study included 95 participants, comprising 55 individuals with substance use disorder (SUD) and 40 age- and sex-matched healthy controls. Participants in the study group were recruited from individuals presenting to psychiatry/addiction services for routine (non-acute) clinical evaluation.

### 2.3. Study Group Characteristics

Participants in the study group had a minimum substance use duration of at least one year and were evaluated during the active phase of SUD under clinically stable conditions, excluding acute intoxication and severe withdrawal states. Substance use patterns were recorded and categorized as single-substance or polysubstance use. Blood samples were obtained from individuals whose last substance use occurred between 2 weeks and 2 months prior to assessment, reflecting subacute or chronic exposure.

Clinical evaluation was conducted by experienced psychiatrists using DSM-5 criteria and the structured SCID-5-CV interview. Diagnostic information was supported by clinical records and collateral information when available.

Due to the retrospective design, standardized measures of illness severity and detailed quantitative data on substance use characteristics (e.g., frequency and amount) were not consistently available.

### 2.4. Control Group

The control group consisted of age- and sex-matched healthy volunteers recruited from individuals undergoing routine health evaluations. All participants were screened through clinical assessment, and those with a history of substance use disorder, psychiatric illness, chronic systemic disease, acute or inflammatory conditions, active infection, hematological disorders, alcohol or tobacco use, or use of medications affecting hematological or coagulation parameters were excluded to ensure comparability.

### 2.5. Exclusion Criteria

Individuals with chronic systemic diseases, acute medical or inflammatory conditions, active infections, hematological disorders, major psychiatric comorbidities, alcohol use, or medications affecting hematological or coagulation parameters were excluded from the study. Participants under the age of 18 were not included.

### 2.6. Statistical Analysis

Statistical analyses were performed using IBM SPSS Statistics version 27.0 (IBM Corp., Armonk, NY, USA). Descriptive statistics were used to summarize demographic and laboratory data. The normality of data distribution was assessed using the Shapiro–Wilk test. Continuous variables were expressed as mean ± standard deviation (SD) for normally distributed data, and median with interquartile range (IQR) for non-normally distributed data. Comparisons between the study and control groups were performed using the independent samples *t*-test for normally distributed variables and the Mann–Whitney U test for non-normally distributed variables. Categorical variables were analyzed using the chi-square (χ^2^) test. Effect sizes (Cohen’s d) were calculated using pooled standard deviations to ensure accurate estimation and consistency with the statistical test results. Effect sizes were interpreted as small (0.20–0.49), moderate (0.50–0.79), and large (≥0.80). Given the number of comparisons performed across multiple hematological parameters, no formal correction for multiple testing was applied; therefore, the results should be interpreted as exploratory. A *p*-value < 0.05 was considered statistically significant.

## 3. Results

### 3.1. Demographic Characteristics

The demographic characteristics of the participants were compared between the study groups. In the study group, 90.9% of the participants were male (*n* = 50) and 9.1% were female (*n* = 5), whereas in the control group 85.0% were male (*n* = 34) and 15.0% were female (*n* = 6). No statistically significant difference was observed between the groups in terms of gender distribution (*p* = 0.518).

The mean age of participants in the study group was 27.62 ± 6.69 years (range: 19–48), while the mean age in the control group was 27.73 ± 8.04 years (range: 18–57). Independent samples *t*-test analysis revealed no statistically significant difference between the groups regarding age (*p* = 0.944). The demographic characteristics of the participants are summarized in Table 1.

### 3.2. Substance Use Characteristics

Among the individuals in the study group, 25 participants reported single-substance use, whereas 30 participants reported polysubstance use. The distribution of substances used by patients is presented in Table 2.

### 3.3. Hematological Parameters

When hematological parameters were compared between the study and control groups, several significant differences were observed. The WBC levels were significantly higher in the study group than in the control group [9.56 (2.91) vs. 6.24 (1.94); *p* = 0.001]. Similarly, platelet (PLT) levels were significantly elevated in the study group compared with the controls [301.00 (95.00) vs. 243.50 (83.50); *p* = 0.005]. The mean corpuscular volume (MCV) was also significantly higher in the study group [89.70 (7.70) vs. 86.50 (7.85); *p* = 0.017], whereas the mean corpuscular hemoglobin concentration (MCHC) was significantly higher in the control group [33.20 (1.70) vs. 32.30 (1.70); *p* = 0.001]. Red cell distribution width parameters were elevated in the study group. Both RDW-CV [13.40 (0.90) vs. 13.10 (0.95); *p* = 0.032] and RDW-SD [44.20 (3.60) vs. 43.20 (3.07); *p* = 0.020] were significantly higher in this group. PCT was also significantly higher in the study group (0.30 ± 0.06 vs. 0.27 ± 0.05; *p* = 0.011). In contrast, PDW and platelet-large cell ratio (P-LCR) were significantly higher in the control group (PDW: 16.27 ± 0.41 vs. 16.07 ± 0.34; *p* = 0.015, P-LCR: 28.50 ± 8.03 vs. 25.33 ± 6.51; *p* = 0.036).

Neutrophil percentage and absolute neutrophil counts were significantly elevated in the study group (NEU%: 62.56 ± 10.92 vs. 55.99 ± 8.07; *p* = 0.002, NEU: 5.99 ± 2.15 vs. 3.76 ± 1.05; *p* = 0.001). Conversely, lymphocyte percentage was higher in the control group [35.65 (9.23) vs. 28.80 (15.20); *p* = 0.002], while absolute lymphocyte counts were higher in the study group (2.55 ± 0.86 vs. 2.27 ± 0.74; *p* = 0.004). Basophil percentage was higher in the control group [0.50 (0.27) vs. 0.40 (0.02); *p* = 0.014], whereas absolute monocyte counts were significantly higher in the study group (0.61 ± 0.20 vs. 0.46 ± 0.18; *p* = 0.001).

Inflammatory ratios were also significantly elevated in the study group. The NLR was significantly higher [2.20 (1.84) vs. 1.53 (0.68); *p* = 0.002], as was the monocyte-to-lymphocyte ratio (MLR) [0.22 (0.13) vs. 0.19 (0.07); *p* = 0.018]. No statistically significant differences were detected between groups for RBC, HGB, HCT, MCH, MPV, P-LCC, EOS%, EOS, MON%, EOS/LYM, or BAS/LYM (*p* > 0.05). Hemogram parameters are presented in Table 3.

### 3.4. Effect Size Analysis

Effect size analyses revealed very large effects for WBC (d = 1.30) and absolute neutrophil count (NEU; d = 1.77), suggesting that the observed differences were not only statistically significant but may also be clinically relevant. Moderate effect sizes (0.5–0.8) were observed for PLT, RDW-SD, MON, LYM, NEU%, P-LCR, NLR, and MLR, supporting the potential clinical relevance of these findings. In contrast, RDW-CV, MCV, PDW, MCHC, and BAS% demonstrated small effect sizes (0.2–0.5), indicating limited clinical impact despite statistical significance. For variables without statistically significant differences, effect sizes were generally very small (<0.2), further supporting the absence of meaningful group differences.

### 3.5. Coagulation Parameters

When coagulation parameters were compared between the study and control groups, no statistically significant differences were observed in activated partial thromboplastin time (APTT) (26.06 ± 3.10 vs. 25.67 ± 2.25; *p* = 0.497) or prothrombin activity (PT%) [98.60 (20.30) vs. 94.45 (18.28); *p* = 0.459]. However, the PT-INR levels were significantly higher in the control group compared with the study group (1.08 ± 0.05 vs. 1.04 ± 0.09; *p* = 0.029). Effect size analysis revealed a very large effect size for APTT (d = 2.77), although the difference did not reach statistical significance, possibly reflecting variance differences between groups. Despite statistical significance for PT-INR, the effect size was very small (d = 0.08), suggesting limited clinical relevance. PT (%) demonstrated a large, calculated effect size (d = 15.3), likely influenced by outliers and inconsistent with the statistical test results. Overall, the coagulation time parameters did not show clinically meaningful differences between groups. These results are summarized in Table 4.

### 3.6. Fibrinolytic Parameters

Among the fibrinolytic markers, the D-dimer levels were significantly higher in the study group compared with the control group [245.66 (143.40) vs. 163.57 (120.11); *p* = 0.001], suggesting increased fibrin degradation and enhanced thrombotic activity in the study group. In contrast, no statistically significant difference was observed in fibrinogen levels [224.77 (69.80) vs. 221.30 (54.10); *p* = 0.295]. Effect size analysis demonstrated a moderate effect size for D-dimer (d = 0.61), supporting its potential clinical relevance. In contrast, fibrinogen showed a small effect size (d = 0.25), indicating minimal clinical difference between groups. These findings are presented in Table 5.

### 3.7. Single vs. Polysubstance Use Analysis

Within the patient group, hematocrit (HCT) levels were significantly higher in individuals using multiple substances (47.93 ± 3.68) compared with those using a single substance (45.55 ± 4.58; *p* = 0.037). Similarly, platelet-large cell count (P-LCC) was significantly elevated in the polysubstance use group (80.77 ± 19.74 vs. 67.44 ± 14.34; *p* = 0.007), suggesting that increased substance exposure may influence platelet morphology and large platelet counts. No statistically significant differences were observed for the other hematological or coagulation parameters (*p* > 0.05). Effect size analysis demonstrated a large effect for HCT (d = 4.11) and an extremely large effect for P-LCC (d = 17.51), indicating that polysubstance use may have clinically meaningful effects on HCT levels and large platelet concentrations. These findings are summarized in Table 6.

**Figure 1:**
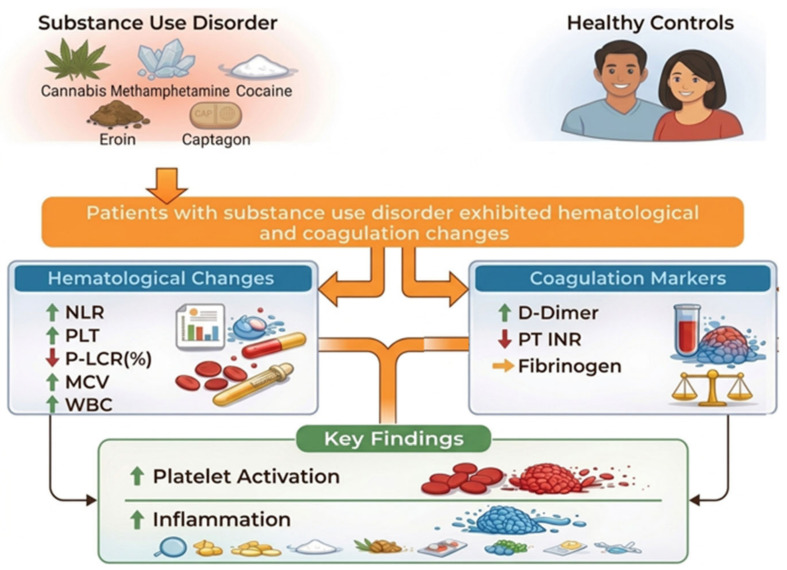
Comparative overview of the hematological indices and coagulation markers between individuals with substance use disorder (SUD) and healthy controls.

Our analysis revealed significant hematological and coagulation alterations in patients with SUD compared to the healthy controls. Specifically, the SUD group exhibited elevated systemic inflammatory markers, characterized by a higher NLR. Regarding platelet indices, the total platelet count (PLT) was significantly higher in the SUD group, whereas the platelet-large cell ratio (P-LCR) was higher in the control group and platelet-large cell count (P-LCC) did not differ significantly between groups. Furthermore, the D-dimer levels were elevated in individuals with SUD, suggesting increased fibrinolytic activity. However, the fibrinogen levels did not show a statistically significant difference between groups. Figure 1 provides a descriptive summary of the hematological and coagulation alterations observed in the study group compared with the controls, without implying a direct mechanistic relationship between these parameters.

Given the number of comparisons performed, no formal correction for multiple testing was applied; therefore, the results should be interpreted as exploratory.

The figure illustrates the hematological and coagulation alterations observed in the study group (SUD), with healthy controls serving as the reference group. The diagram is descriptive and does not imply causality or direct mechanistic relationships between parameters.

## 4. Discussion

The present study investigated CBC-derived inflammatory indices in individuals with SUD and compared them with healthy controls. The main findings of this study indicate that patients with SUD exhibited significantly altered hematological inflammatory parameters, suggesting the presence of systemic inflammatory activation. Inflammatory indices derived from routine CBC parameters were elevated in the SUD group compared with the controls, supporting the hypothesis that SUDs are associated with an enhanced inflammatory response. These findings contribute to the growing body of evidence indicating that inflammatory mechanisms may play an important role in the pathophysiology of addiction. The observed alterations in hematological inflammatory markers may reflect the activation of immune and neuroimmune pathways associated with chronic substance exposure. Psychoactive substances have been shown to induce neuroinflammatory responses through mechanisms involving microglial activation, oxidative stress, and cytokine dysregulation, which may ultimately contribute to the development and maintenance of addictive behaviors. Therefore, the elevated inflammatory indices observed in the present study may represent peripheral manifestations of these underlying neurobiological processes. Unlike prior studies evaluating individual biomarkers, the present study integrates hematological inflammatory indices with platelet morphology-related parameters and coagulation/fibrinolytic markers, offering a more comprehensive perspective on systemic alterations associated with SUD. These findings should be interpreted with caution, as the analyses are exploratory in nature and not adjusted for multiple comparisons.

Among the evaluated parameters, the NLR and PLR are considered reliable indicators of systemic inflammatory balance and have been increasingly investigated as accessible inflammatory biomarkers in various medical conditions. These indices reflect the interplay between innate immune activation and adaptive immune regulation, with elevated neutrophil and platelet counts representing inflammatory activation, while reduced lymphocyte levels may indicate physiological stress and impaired immune regulation. Previous studies have demonstrated that NLR and PLR are associated with several psychiatric and neurological disorders, including major depressive disorder, schizophrenia, bipolar disorder, and neurodegenerative diseases, suggesting that systemic inflammation may contribute to the pathophysiology of these conditions [8,17,24]. In the context of SUDs, emerging evidence indicates that chronic exposure to psychoactive substances may activate neuroimmune pathways, leading to peripheral inflammatory alterations detectable through hematological markers [9]. Moreover, substances such as alcohol, opioids, and stimulants have been shown to induce oxidative stress, cytokine dysregulation, and microglial activation, which may contribute to persistent inflammatory responses in both the central nervous system and in peripheral circulation [15]. Consistent with these mechanisms, several recent studies have reported elevated NLR and PLR values in individuals with SUD, supporting the hypothesis that addiction-related neurobiological processes are closely linked to systemic inflammatory activation [2,22,25].

Consistent with these findings, several recent studies have reported alterations in hematological inflammatory parameters among individuals with SUDs. For instance, studies examining patients with methamphetamine and synthetic cannabinoid use disorder demonstrated significantly elevated NLR values compared with healthy controls, suggesting the presence of increased systemic inflammatory activity in these populations [10]. Similarly, investigations focusing on alcohol and cocaine use disorders have reported higher levels of inflammatory hematological markers, further supporting the hypothesis that chronic substance exposure is associated with immune system activation and inflammatory dysregulation [19]. These observations align with the neurobiological framework of addiction, which propose that persistent exposure to psychoactive substances leads to neuroimmune activation, oxidative stress, and the dysregulation of cytokine signaling pathways [26].

Our findings can be interpreted considering previous studies examining hematological alterations in individuals with SUDs. Bani-Ahmad et al. [27] evaluated hematological parameters in 30 male patients with chronic polysubstance use disorder and 30 healthy controls who had used multiple substances, including nicotine, heroin, methamphetamine, fenethylline, cannabis, and misused prescription drugs, for an average of 4.1 ± 3.4 years. The authors reported significant differences in erythrocyte-related parameters, including red blood cell count, hematocrit, MCH, and MCHC, as well as higher neutrophil and lower monocyte counts in patients compared with the controls. Similarly, in the present study, MCV was significantly higher in the SUD group, whereas the MCHC levels were higher in the controls. However, unlike the findings of Bani-Ahmad et al. [27], monocyte levels (MON) were significantly higher in our patient group. This discrepancy may be related to differences in substance profiles, duration of exposure, or demographic and clinical characteristics between the study populations.

The discrepancy in monocyte levels between studies may reflect the complex and heterogeneous immune responses associated with SUDs. Monocytes are key components of the innate immune system and play an important role in systemic inflammatory regulation [28]. Chronic exposure to psychoactive substances has been shown to activate peripheral immune pathways and promote the release of pro-inflammatory cytokines, which may influence monocyte production, activation, and trafficking. In addition, different substances may exert distinct immunomodulatory effects; for example, stimulants, opioids, and synthetic cannabinoids have been reported to differentially affect immune signaling pathways and hematopoietic activity. Variations in substance type, duration of exposure, severity of dependence, and patterns of polysubstance use may therefore contribute to divergent monocyte responses across study populations. Furthermore, substance-induced oxidative stress and neuroimmune activation may alter monocyte migration between peripheral blood and tissues, potentially leading to variability in circulating monocyte levels observed in patients with SUD [8,9,15,29].

Alterations in coagulation parameters observed in the present study may also reflect the systemic inflammatory state associated with SUDs. Increasing evidence suggests that inflammation and coagulation pathways are closely interconnected, with inflammatory mediators capable of activating the coagulation cascade and promoting a prothrombotic state. Chronic exposure to psychoactive substances may contribute to endothelial dysfunction, oxidative stress, and platelet activation, all of which may influence coagulation profiles. Previous studies have demonstrated that substance use, particularly involving stimulants and opioids, can alter hemostatic balance and increase the risk of thrombotic events through inflammatory and vascular mechanisms. Therefore, the changes in coagulation parameters observed in our study may represent an additional manifestation of systemic inflammatory dysregulation in individuals with SUD [12,13,15].

Substance use is known to influence the coagulation system through multiple biological mechanisms. A review by Carter et al. [30] reported that cannabis use may influence platelet function by modulating platelet activation and aggregation processes. In addition, previous studies suggest that psychoactive substances can differentially affect liver function, highlighting the importance of considering substance-specific effects when evaluating clinical populations [31,32]. Bardakçı et al. [33] evaluated coagulation and liver-related parameters in individuals with SUDs compared with healthy controls. Their study demonstrated significant alterations in several coagulation markers, suggesting that chronic substance exposure may influence hemostatic balance through inflammatory and metabolic mechanisms. These findings are generally consistent with the results of the present study, which also identified alterations in coagulation-related parameters in patients with SUD. Together, these observations support the growing evidence that chronic substance use may contribute to systemic inflammatory activation and disturbances in coagulation pathways. Such alterations may reflect endothelial dysfunction, platelet activation, and inflammatory responses associated with long-term substance exposure. Sharma et al. [14] also found that cocaine use, a stimulant like methamphetamine, contributes to prothrombotic condition. Levendal and Frost [34] investigated the in vivo effects of *Cannabis sativa* extract on blood coagulation and metabolic parameters in experimental animal models and reported that cannabis exposure may influence hemostatic processes. These findings suggest that certain psychoactive substances can modulate coagulation pathways through metabolic and inflammatory mechanisms. In line with these observations, the alterations in coagulation-related parameters identified in the present study may reflect the impact of chronic substance exposure on hemostatic balance in individuals with SUD. Previous studies have reported no significant association between opioid use and coagulation parameters such as PT, aPTT, and INR [35,36]. Consistent with these findings, the present study did not demonstrate clinically meaningful differences in coagulation time parameters between individuals with SUD and healthy controls. Although a statistically significant difference was observed for PT-INR, the very small effect size suggests limited clinical relevance. In contrast, the significantly elevated D-dimer levels in the study group may indicate increased fibrinolytic activity and potential thrombotic alterations associated with chronic substance exposure. Bardakcı et al. [33] reported that PLT levels were lower in individuals with alcohol use compared with polysubstance and methamphetamine users, although no significant difference was observed relative to healthy controls. Previous studies have suggested that chronic alcohol consumption may reduce platelet counts and impair platelet aggregation, whereas acute exposure may transiently enhance platelet activation [37,38]. In the present study, platelet-large cell count (P-LCC) was significantly higher in the polysubstance use group, indicating that increased substance exposure may influence platelet morphology and the proportion of large platelets. These findings suggest that different substances may exert distinct effects on platelet dynamics and hemostatic regulation. SUD involves not only the activation or inactivation of cells such as leukocytes, but also a severe disruption of the body’s clotting mechanism (hemostasis), both through direct chemical effects and indirect damage to the liver and vascular system. This leads to inconsistent results in coagulation tests.

In the present study, although PT-INR demonstrated statistical significance (*p* = 0.029), the effect size was very small (d = 0.08), suggesting limited clinical relevance. Therefore, this finding should be interpreted with caution and not considered as a strong indicator of coagulation imbalance on its own. These results highlight the importance of interpreting statistically significant findings together with effect sizes to avoid the overestimation of clinical impact.

Previous studies have reported associations between systemic inflammation, coagulation markers, and neuroinflammatory processes. For instance, fibrinogen has been suggested to be associated with neuroinflammatory responses under certain pathological conditions [39]. Similarly, recent studies indicate that combining hematological indices such as NLR/PLR with D-dimer may provide a broader representation of systemic inflammatory and fibrinolytic activity in different clinical settings [40].

In addition, the liver plays a central role in the synthesis of coagulation factors and acute-phase reactants, and systemic metabolic or inflammatory alterations have been reported to be associated with changes in coagulation and immune profiles [41]. Some studies also suggest that integrated approaches combining hematological indices and clinical parameters may reflect systemic inflammatory burden [42]. Furthermore, the concept of “thrombo-inflammation” has been proposed to describe the interaction between inflammatory and coagulation pathways, including platelet and leukocyte activity [43].

However, in the context of the present study, these findings should be interpreted as associations rather than evidence of direct mechanistic or causal relationships. Given the cross-sectional design and the absence of molecular or neurobiological markers, interpretations related to neuroinflammation or thrombo-inflammatory mechanisms remain speculative and beyond the scope of the current data.

### 4.1. Clinical Implications

The present findings provide additional evidence that SUDs may be associated with systemic inflammatory activation and alterations in hematological and coagulation-related parameters. From a clinical perspective, laboratory markers derived from routine complete blood count and coagulation tests, including inflammatory ratios and fibrinolytic indicators such as D-dimer, may serve as accessible and cost-effective tools for evaluating biological alterations in individuals with SUD. Although these markers are not specific diagnostic indicators, their assessment may help clinicians better understand the systemic effects of chronic substance exposure and may contribute to the identification of patients who require closer clinical monitoring. Furthermore, the integration of hematological and coagulation markers with clinical assessments may provide additional insights into the inflammatory and vascular consequences of long-term substance use. This integrative approach may provide added clinical value by simultaneously capturing the inflammatory, platelet-related, and hemostatic pathways, which are often investigated separately in the existing literature.

### 4.2. Strengths and Limitations

This study has several strengths. First, it simultaneously evaluated hematological inflammatory indices, platelet-related parameters, and coagulation markers in individuals with SUD, allowing for a more comprehensive assessment of systemic inflammatory and hemostatic alterations. Second, the inclusion of a healthy control group enabled a direct comparison of laboratory parameters between patients and individuals without SUD. Third, the use of routinely available laboratory markers increases the potential clinical applicability of the findings.

However, several limitations should be acknowledged. The cross-sectional design precludes causal inference, and the relatively small sample size, along with sex imbalance, may limit the generalizability of the findings. The heterogeneous composition of the study population, including multiple substance types and polysubstance use, restricts substance-specific interpretation of the observed hematological and coagulation alterations. In addition, important potential confounders, including nicotine use, body mass index, nutritional status, and the duration and severity of substance use, could not be systematically assessed or adjusted for, which may have influenced the results. Due to the retrospective design, detailed quantitative data on substance use characteristics (e.g., frequency and amount) and standardized measures of illness severity were not consistently available, limiting comprehensive clinical characterization. Given the multiple comparisons performed, no formal correction for multiple testing was applied; therefore, the findings should be interpreted as exploratory. Finally, the absence of molecular inflammatory markers limits mechanistic insight into the observed associations.

### 4.3. Future Directions

Future research should aim to include larger and more heterogeneous populations to better clarify the relationship between SUDs and hematological or coagulation markers. Longitudinal studies would be particularly valuable in determining whether these alterations represent consequences of chronic substance exposure or potential biomarkers associated with disease progression. In addition, integrating routine hematological parameters with molecular inflammatory markers, cytokine profiles, and neurobiological indicators may provide a more comprehensive understanding of the biological pathways involved in SUDs. Such multidisciplinary approaches may contribute to the identification of potential biological markers that could support clinical assessment and monitoring of individuals with SUD.

To our knowledge, this study is among the limited number of investigations that simultaneously evaluated hematological inflammatory indices, platelet-related parameters, and coagulation markers in individuals with SUD, providing a more comprehensive perspective on systemic inflammatory and hemostatic alterations associated with chronic substance exposure. From a translation perspective, the assessment of routinely available hematological and coagulation parameters may offer a practical and cost-effective approach for detecting systemic inflammatory and hemostatic disturbances in individuals with SUD, and may contribute to improved clinical monitoring in this population.

In conclusion, the present study demonstrated that individuals with SUD exhibit alterations in hematological inflammatory indices, platelet-related parameters, and fibrinolytic markers compared with healthy controls. These findings support the growing body of evidence suggesting that systemic inflammation and hemostatic dysregulation may be associated with the biological processes underlying SUD. Routine laboratory markers derived from CBC and coagulation tests may reflect these systemic alterations; however, their clinical applicability as biomarkers remains to be established. Given the cross-sectional design and absence of diagnostic or predictive analyses, these findings should be considered exploratory and hypothesis-generating. Collectively, our findings suggest that routinely available hematological and coagulation parameters may serve as accessible indicators of systemic alterations in SUD, warranting further investigation in larger, longitudinal, and validation studies.

## Figures and Tables

**Table 1:** Distribution of demographic characteristics by group.

Variable	Category	Group				*p* ^k^
		Study Group (*n* = 55) *n* (%)		Control Group (*n* = 40) *n* (%)		
		*n*	%	*n*	%	
**Sex**	Female	5	9.09	6	15.00	0.518
	Male	50	90.91	34	85.00	
**Variable**		Mean ± SD	Min–Max	Mean ± SD	Min–Max	*p* ^t^
**Age (Year)**		27.62 ± 6.69	19–48	27.73 ± 8.04	18–57	0.944

*p* < 0.05; SD: standard deviation. ^k^ = Chi-square test (used for categorical variables) and ^t^ = independent samples *t*-test (used continuous variables). **Min:** Minimum; **Max:** Maximum.

**Table 2:** Demographic characteristics and substance use profiles of the patient cohort.

Patient ID	Age	Gender	Substance Used (Past 1 Year of Use, Last Use Between 2 Weeks–2 Months Ago)	Single-Substance Polysubstance
1	33	Female	Heroin	Single
2	27	Male	Methamphetamine, Cannabis	Poly
3	20	Male	Heroin	Single
4	33	Male	Heroin	Single
5	26	Male	Methamphetamine, Heroin	Poly
6	38	Male	Methamphetamine, Cannabis	Poly
7	24	Male	Cannabis	Single
8	26	Male	Heroin	Single
9	38	Male	Heroin	Single
10	24	Male	Methamphetamine, Captagon	Poly
11	19	Male	Methamphetamine	Single
12	30	Male	Cannabis, Methamphetamine	Poly
13	26	Male	Methamphetamine	Single
14	21	Male	Cannabis	Single
15	24	Male	Cannabis	Single
16	30	Male	Heroin	Single
17	29	Male	Cannabis	Single
18	43	Male	Methamphetamine, Ecstasy, Cannabis	Poly
19	29	Male	Methamphetamine, Cannabis	Poly
20	21	Female	Methamphetamine	Single
21	48	Male	Methamphetamine, Cannabis, Ecstasy	Poly
22	20	Male	Heroin, Cannabis	Poly
23	33	Male	Methamphetamine, Ecstasy, Cannabis	Poly
24	23	Male	Methamphetamine, Ecstasy, Cannabis	Poly
25	33	Male	Methamphetamine, Cannabis	Poly
26	21	Male	Methamphetamine, Cannabis	Poly
27	20	Male	Methamphetamine, Cannabis	Poly
28	20	Male	Methamphetamine	Single
29	28	Male	Methamphetamine, Cannabis	Poly
30	27	Male	Methamphetamine, Cannabis	Poly
31	22	Male	Cannabis, Bonsai	Poly
32	24	Male	Methamphetamine, Ecstasy, Cannabis	Poly
33	32	Male	Methamphetamine, Heroin	Poly
34	28	Male	Methamphetamine, Cannabis	Poly
35	23	Male	Methamphetamine, Benzodiazepine	Poly
36	32	Male	Methamphetamine, Cannabis	Poly
37	24	Male	Cocaine	Single
38	19	Female	Methamphetamine	Single
39	34	Male	Methamphetamine, Cannabis	Poly
40	26	Male	Methamphetamine, Heroin	Poly
41	40	Male	Ecstasy	Single
42	28	Male	Methamphetamine, Heroin	Poly
43	45	Male	Cannabis	Single
44	27	Male	Cannabis	Single
45	30	Male	Methamphetamine, Ecstasy, Cannabis	Poly
46	26	Male	Cannabis	Single
47	25	Male	Methamphetamine	Single
48	34	Male	Methamphetamine	Single
49	25	Male	Cannabis	Single
50	21	Male	Cannabis	Single
51	28	Male	Methamphetamine, Ecstasy, Cannabis	Poly
52	26	Male	Cannabis, Ecstasy	Poly
53	21	Female	Methamphetamine	Single
54	25	Female	Methamphetamine, Ecstasy	Poly
55	20	Male	Methamphetamine, Ecstasy, Bonsai	Poly

**Table 3:** Comparison of complete blood count (CBC) parameters between the study and control groups.

Variable	Group				*p*	Effect Size (Cohen’s d)
	Study Group (*n* = 55)		Control Group (*n* = 40)			
	Mean ± SD	Median–IQR	Mean ± SD	Median–IQR		
**WBC (10^3^/µL)**	9.37 ± 2.36	9.56–2.91	6.69 ± 1.56	6.24–1.94	**0.001** * ^u^	1.30
**RBC (10^6^/µL)**	5.26 ± 0.52	5.24–0.53	5.35 ± 0.53	5.37–0.59	0.393 ^t^	0.17
**HGB (g/dL)**	15.12 ± 1.43	15.10–1.60	15.47 ± 1.55	15.80–1.52	0.258 ^t^	0.23
**HCT (%)**	46.85 ± 4.25	47.00–5.70	46.17 ± 4.68	46.40–5.80	0.464 ^t^	0.15
**PLT (10^3^/µL)**	304.65 ± 76.77	301.00–95.00	261.78 ± 63.46	243.50–83.50	**0.005** * ^u^	0.60
**MCV (fL)**	89.33 ± 6.42	89.70–7.70	86.46 ± 6.63	86.50–7.85	**0.017** * ^u^	0.44
**MCH (pg)**	28.85 ± 2.26	29.50–2.70	28.97 ± 2.29	29.45–2.00	0.985 ^u^	0.05
**MCHC (g/dL)**	32.29 ± 1.04	32.30–1.70	33.53 ± 1.38	33.20–1.70	**0.001** * ^u^	1.04
**RDW-CV (%)**	13.66 ± 1.25	13.40–0.90	13.27 ± 1.01	13.10–0.95	**0.032** * ^u^	0.34
**RDW-SD (fL)**	44.86 ± 4.10	44.20–3.60	43.00 ± 2.53	43.20–3.07	**0.020** * ^u^	0.54
**MPV (fL)**	9.87 ± 0.94	10.10–1.40	10.30 ± 1.18	10.20–1.80	0.052 ^t^	0.41
**PCT (%)**	0.30 ± 0.06	0.29–0.08	0.27 ± 0.05	0.25–0.09	**0.011** * ^t^	0.55
**PDW (fL)**	16.07 ± 0.34	16.10–0.50	16.27 ± 0.41	16.20–0.70	**0.015** * ^t^	0.54
**P-LCC (fL)**	74.71 ± 18.59	73.00–29.00	71.75 ± 17.12	71.00–20.25	0.431 ^t^	0.17
**P-LCR (%)**	25.33 ± 6.51	27.00–9.90	28.50 ± 8.03	28.30–13.13	**0.036** * ^t^	0.43
**NEU% (%)**	62.56 ± 10.92	62.50–16.90	55.99 ± 8.07	54.25–9.65	**0.002** * ^t^	0.66
**NEU (10^3^/µL)**	5.99 ± 2.15	5.83–2.92	3.76 ± 1.05	3.66–1.22	**0.001** * ^t^	1.25
**LYM (%)**	28.52 ± 10.15	28.80–15.20	34.08 ± 7.28	35.65–9.23	**0.002** * ^u^	0.62
**LYM (10^3^/µL)**	2.55 ± 0.86	2.44–1.30	2.27 ± 0.74	2.07–0.84	**0.004** * ^t^	0.35
**EOS (%)**	2.04 ± 1.29	2.10–1.90	2.58 ± 1.81	2.35–1.50	0.144 ^u^	0.35
**EOS (10^3^/µL)**	0.17 ± 0.14	0.13–0.20	0.17 ± 0.11	0.15–0.14	0.566 ^u^	0.02
**BAS (10^3^/µL)**	0.05 ± 0.05	0.04–0.03	0.03 ± 0.01	0.03–0.02	0.066 ^u^	0.50
**BAS (%)**	0.40 ± 0.17	0.40–0.02	0.53 ± 0.26	0.50–0.27	**0.014** * ^u^	0.59
**MON (%)**	6.48 ± 1.31	6.50–1.80	6.82 ± 1.82	6.60–2.10	0.632 ^u^	0.22
**MON (10^3^/µL)**	0.61 ± 0.20	0.56–0.26	0.46 ± 0.18	0.43–0.23	**0.001** * ^t^	0.79
**NLR (NEU/LYM)**	2.82 ± 2.23	2.20–1.84	1.84 ± 1.06	1.53–0.68	**0.002** * ^u^	0.55
**ELR (EOS/LYM)**	0.08 ± 0.09	0.07–0.05	0.08 ± 0.05	0.07–0.06	0.623 ^u^	0.05 (≈0)
**BLR (BAS/LYM)**	0.02 ± 0.02	0.01–0.01	0.02 ± 0.01	0.02–0.01	0.342 ^u^	0.05 (≈0)
**MLR (MON/LYM)**	0.27 ± 0.18	0.22–0.13	0.21 ± 0.10	0.19–0.07	**0.018** * ^u^	0.40

SI units were used for all hematological parameters. NLR, ELR, BLR, and MLR were calculated as numerical ratios. **SD:** Standard Deviation; **WBC:** White Blood Cell Count; **RBC:** Red Blood Cell Count; **HGB**: Hemoglobin; **HCT:** Hematocrit; **PLT:** Platelet Count; **MCV:** Mean Corpuscular Volume; **MCH:** Mean Corpuscular Hemoglobin; **MCHC:** Mean Corpuscular Hemoglobin Concentration; **RDW-CV:** Red Cell Distribution Width-Coefficient of Variation; **RDW-SD:** Red Cell Distribution Width-Standard Deviation; **MPV:** Mean Platelet Volume; **PCT:** Plateletcrit; **PDW:** Platelet Distribution Width; **P-LCC**: Platelet-Large Cell Count; **P-LCR:** Platelet-Large Cell Ratio; **NEU:** Neutrophil; **LYM:** Lymphocyte; **EOS**: Eosinophil; **BAS**: Basophil; **MON**: Monocyte; **NLR:** Neutrophil-to-Lymphocyte Ratio; **ELR:** Eosinophil-to-Lymphocyte Ratio; **BLR:** Basophil-to-Lymphocyte Ratio; **MLR:** Monocyte-to-Lymphocyte Ratio; **IQR:** Interquartile Range; *p*: Statistical Significance Value; * *p* < 0.05; ^t^: Student’s *t*-Test; ^u^: Mann–Whitney U Test.

**Table 4:** Distribution of coagulation parameters by group.

Variable	Group				*p*	Effect Size (Cohen’s d)
	Study Group (*n* = 55)		Control Group (*n* = 40)			
	Mean ± SD	Median–IQR	Mean ± SD	Median–IQR		
**aPTT (s)**	26.06 ± 3.10	25.70–4.20	25.67 ± 2.25	25.65–2.93	0.497 ^t^	2.77
**PT INR (s)**	1.04 ± 0.09	1.04–0.15	1.08 ± 0.05	1.07–0.08	**0.029** * ^t^	0.08
**PT (%)**	97.82 ± 17.37	98.60–20.30	95.46 ± 11.85	94.45–18.28	0.459 ^u^	15.3

* *p* < 0.05 was considered statistically significant. **aPTT:** Activated Partial Thromboplastin Time; **PT:** Prothrombin Time; **INR:** International Normalized Ratio; **SD:** Standard Deviation; **IQR:** Interquartile Range; *p*: Statistical Significance Value; ^t^: Independent Samples *t*-Test; ^u^: Mann–Whitney U Test.

**Table 5:** Distribution of coagulation and hemostasis parameters by group.

Variable	Group				*p*	Effect Size (Cohen’s d)
	Study Group (*n* = 55)		Control Group (*n* = 40)			
	Mean ± SD	Median–IQR	Mean ± SD	Median–IQR		
**Fibrinogen (mg/dL)**	241.05 ± 55.11	224.77–69.80	228.28 ± 42.23	221.30–54.10	0.295 ^u^	0.25
**D-Dimer (ng/mL)**	263.99 ± 155.09	245.66–143.40	183.91 ± 84.49	163.57–120.11	**0.001** * ^u^	0.61

* *p* < 0.05; ^u^ = Mann–Whitney U Testi.

**Table 6:** Differentiation of hematological parameters according to the number of substances used.

Variable	Substance Use				*p*	Effect Size (Cohen’s d)
	Single Substance Use (*n* = 25)		Polysubstance Use (*n* = 30)			
	Mean	SD	Mean	SD		
**WBC (10^3^/µL)**	9.67	2.21	9.12	2.49	0.395	2.37
**RBC (10^6^/µL)**	5.17	0.50	5.34	0.52	0.244	0.51
**HGB (g/dL)**	14.76	1.55	15.43	1.27	0.084	1.41
**HCT (%)**	45.55	4.58	47.93	3.68	**0.037** *	4.11
**PLT (10^3^/µL)**	297.28	84.80	310.80	70.27	0.521	77.18
**MCV (fL)**	88.28	6.39	90.21	6.42	0.269	6.40
**MCH (pg)**	28.62	2.39	29.03	2.17	0.504	2.27
**MCHC (g/dL)**	32.40	1.07	32.19	1.02	0.446	1.05
**RDW-CV (%)**	13.47	1.07	13.82	1.38	0.309	1.25
**RDW-SD (fL)**	43.62	3.19	45.89	4.52	0.051	3.98
**MPV (fL)**	9.64	0.86	10.07	0.97	0.091	0.92
**PCT (%)**	0.28	0.07	0.31	0.06	0.109	0.06
**PDW (fL)**	16.08	0.41	16.07	0.27	0.948	0.34
**P-LCC (fL)**	67.44	14.34	80.77	19.74	**0.007** *	17.51
**P-LCR (%)**	23.77	6.08	26.63	6.67	0.105	19.73
**NEU% (%)**	61.41	11.85	63.53	10.18	0.479	6.41
**NEU (10^3^/µL)**	6.08	2.18	5.92	2.17	0.792	10.97
**LYM (%)**	29.74	10.99	27.50	9.46	0.419	2.17
**LYM (10^3^/µL)**	2.75	0.92	2.38	0.79	0.123	10.18
**EOS (%)**	1.97	1.32	2.09	1.29	0.724	0.85
**EOS (10^3^/µL)**	0.16	0.14	0.18	0.14	0.508	1.30
**BAS (10^3^/µL)**	0.06	0.07	0.04	0.02	0.266	0.14
**BAS (%)**	0.37	0.19	0.42	0.15	0.228	0.05
**MON (%)**	6.51	1.37	6.46	1.27	0.885	0.17
**MON (10^3^/µL)**	0.63	0.21	0.59	0.20	0.422	0.20
**NLR (NEU/LYM)**	2.55	1.47	3.05	2.70	0.409	2.23
**ELR (EOS/LYM)**	0.07	0.04	0.09	0.12	0.314	0.09
**BLR (BAS/LYM)**	0.01	0.01	0.02	0.02	0.112	0.02
**MLR (MON/LYM)**	0.26	0.13	0.29	0.22	0.503	0.19
**aPTT (s)**	25.49	2.89	26.53	3.23	0.216	3.08
**PT INR**	1.05	0.10	1.03	0.09	0.491	0.09
**PT (%)**	95.94	21.10	99.39	13.69	0.468	17.44
**Fibrinogen (mg/dL)**	241.53	52.65	240.64	57.98	0.953	55.63
**D-Dimer (ng/mL)**	281.41	199.89	249.47	106.04	0.452	155.71

* *p* < 0.05.

## Data Availability

The original contributions presented in this study are included in the article. Further inquiries can be directed to the corresponding author(s).
